# Examining the mental health of siblings of children with a mental disorder: A scoping review protocol

**DOI:** 10.1371/journal.pone.0274135

**Published:** 2022-09-15

**Authors:** John E. Krzeczkowski, Terrance J. Wade, Brendan F. Andrade, Dillon Browne, Busra Yalcinoz-Ucan, Negin A. Riazi, Elizabeth Yates, Andrea Tagalakis, Karen A. Patte

**Affiliations:** 1 Department of Psychology, York University, Toronto, Ontario, Canada; 2 Department of Health Sciences, Brock University, St. Catharines, Ontario, Canada; 3 Margaret and Wallace McCain Centre for Child, Youth and Family Mental Health, Centre for Addiction and Mental Health, Toronto, Ontario, Canada; 4 Department of Psychiatry, University of Toronto, Toronto, Ontario, Canada; 5 Department of Psychology, University of Waterloo, Waterloo, Ontario, Canada; 6 Brock University Library, Brock University, St. Catharines, Ontario, Canada; 7 Family Partnership Program, Children’s Mental Health Ontario, Toronto, Ontario, Canada; Liv Hospital Gaziantep, TURKEY

## Abstract

**Introduction:**

Mental disorders affect 1 in 5 children having consequences for both the child and their family. Indeed, the siblings of these children are not insulated from these consequences and may experience elevated levels of psychological distress, placing them at increased risk for developing mental disorders. This protocol describes the methodology for a scoping review that will examine how mental disorders in children impact the mental health of their sibling(s). Further, we aim to examine the role of sex, gender, birth order, age of each child, and familial factors (e.g., parent mental illness, family structure), in sibling mental health. The proposed review will also identify resources that aim to support the needs of siblings of children with mental disorders. Taken together, this proposed review aims to take a fundamental step towards determining intervention targets to reduce the transmission of risk between siblings.

**Aim:**

The proposed scoping review aims to address the following questions: i) how do mental disorders (in children <18 years of age) impact the mental health of their sibling(s) (also <18 years of age)? ii) Can we identify resources designed to address the needs of siblings of children with mental disorders?

**Methods:**

We will conduct the proposed scoping review in keeping with the six-stage Arksey and O’Malley Framework and the scoping review methodology provided by the Joanna Briggs Institute. In section i) we outline our research questions. In section ii) we describe our process for identifying studies that examine the mental health of siblings of a child with a mental disorder and studies that provide evidence on resources directed specifically at these siblings. We will search peer-review and grey literature published between 2011 and 2022 from OVID MEDLINE, OVID EMBASE, CINAHL Complete, Proquest Nursing and Allied Health, PsycINFO (via APA platform), Proquest Sociology Collection and Web of Science Core Collection and Proquest Theses and Dissertations. Section iii) describes our process for selecting relevant studies. In sections iv and v, we describe our methods for charting and summarizing relevant data. Finally, in section vi) we describe our integrative knowledge translation plan that aims to include knowledge users in interpretating and translating evidence gathered from the proposed review.

## Introduction

Mental disorders affect 20% of children and have significant adverse effects on the health and well-being of the child, but also their families [1, 2]. The vast majority of research and interventions have been developed for the child experiencing the mental disorder(s) and/or the parents of these children. Although these interventions are extremely important, what is often missing is a clear understanding of the mental health and needs of the siblings of these children. Given that family systems theory posits that the health of one member of a family can have an impact on the well-being of other family members [3], the siblings of children with mental disorders are at increased risk for mental disorders themselves [4]. Furthermore, as parents are required to invest significant time and resources to support their child with the mental disorder, they express concern about their capacity to devote attention to the health and development of the siblings [5]. Despite these concerns, evidence-based programs designed specifically to support the siblings of children with mental disorders are lacking. Without support, there is an increased risk that the needs of siblings will be left unrecognized and/or unmet [5, 6]. Understanding the needs of siblings of children with mental disorders and the scope of potential existing resources directed at these siblings is needed to reduce the risk to siblings and benefit their family’s health.

Three previous studies have reviewed the literature on the emotional/behavioral health of siblings of children with a mental disorder [6–8]. These studies reported greater rates of sibling psychopathology, poorer parent-healthy sibling relationships, as well as elevated levels of delinquent behaviour, anxiety/depression, and social problems. However, these studies reviewed the evidence before 2011. Given this 10-year gap in our current knowledge, it is unclear what has been done recently to investigate this significant issue. Furthermore, while one study described the potential needs of siblings (i.e., coping skills) [5], no studies have attempted to identify and map evidence on potential resources directed specifically at the siblings of children with mental disorders. Therefore, an updated review of this evidence that extends previous work by identifying and describing existing resources for siblings would provide an important step towards investing in siblings’ health.

This protocol outlines the methodology of a proposed scoping review that aims to map the evidence on the mental health of siblings of children with mental disorders and identify potential resources and interventions designed specifically to address the needs of siblings. The scoping review methodology is best positioned to enable us to synthesize data from this heterogeneous evidence base. We will use a developmental psychopathology framework approach to map evidence on the mental health of siblings. This framework will enable us to investigate potential antecedents to psychopathology in siblings, domains of functioning in siblings that are known to cut across risk for multiple forms of psychopathology, and investigate well-being factors that may serve to protect siblings against risk for later mental disorders. Furthermore, our approach will account for the fact that sibling and child age, sex, gender, birth order, and familial risk factors (e.g., parental psychopathology, socioeconomic status, family structure) may influence the transmission of risk between siblings [9]. Accounting for these factors within our mapping framework will provide important contextual evidence on the transmission of risk between siblings.

In summary, the aims of this review are two-fold. First, it will provide a comprehensive understanding of the mental health of siblings of children with mental disorders, including potential risk and protective factors. Second, it will identify and assess the scope of resources and interventions for these siblings. Overall, this proposed review will provide a comprehensive assessment of the effects that child mental illness may have on their sibling(s) and the presence of existing sibling-directed resources. Therefore, it will provide an important foundation for future research aiming to invest in the health of siblings of children with mental disorders.

## Method

### Protocol design

We will conduct the review in keeping with the protocols developed by Arksey and O’Malley [10] and the Joanna Briggs Institute (JBI) manual for evidence synthesis [11]. This methodology involves six stages. In stage 1, we identify the research questions. In stage 2, we describe the process for identifying relevant studies. Stage 3 describes our criteria for selecting studies. In stages 4 and 5, we outline our procedures for charting and summarizing the data, respectively. Finally, we will conduct the optional 6^th^ stage, which involves engaging stakeholders in interpretating and translating the evidence gathered in this review. This scoping review protocol was preregistered with the Open Science Framework (registration DOI: 10.17605/OSF.IO/Q639T). We will report results from the complete scoping review according to the Preferred Reporting Items for Systematic reviews and Meta-Analyses extension for Scoping Reviews (PRISMA-ScR) [12].

### Stage 1: Research questions

We used the JBI Population Concept Context framework [11] to develop our research questions. Research question 1 aims to examine how mental disorders in children (<18 years of age) are associated with the mental health of their sibling(s) (<18 years of age). Nested within this question are three sub-questions: i) does the mental health of siblings differ by relative child age, sex, gender, and birth order? ii) Do familial factors (e.g., parent mental illness, socioeconomic status, family structure) play a role in sibling mental health, and iii) do certain characteristics of the child’s mental illness (e.g., behavioral meltdowns, rages, aggression, depressive episodes) have a particular impact on sibling mental health? For research question 2, we aim to identify resources that are specifically targeted toward siblings of children with mental disorders (<18 years of age). There will be no contextual limits for this review (i.e., racial, sociocultural, geographic constraints), except for language (all studies must be published in English).

### Stage 2: Identify relevant studies

All studies must include an assessment of the mental health of the sibling(s) of children with a mental disorder (<18 years of age and not diagnosed with a physical or mental disorder at baseline). Sibling mental health is broadly defined and can include domains of functioning associated with risk factors for psychopathology/protective factors (e.g., stress, well-being, emotion regulation, affect, or behaviour problems), or assessments of psychopathology symptoms. Studies must also include data on a child in the family indicating that they have either received a mental disorder diagnosis or exceeded a clinical threshold on a measure of psychopathology. For question 2, studies must include a resource or intervention designed specifically to address the needs of the healthy siblings of a child with a mental disorder.

We will exclude studies that examine the effects of child autism or Asperger’s syndrome on the sibling or if a physical health condition is present in either child (i.e., brain injury, cancer, cystic fibrosis, epilepsy) given that this area has received much more attention in the literature (e.g., [13, 14]). Studies that include data on children (either the child with the mental disorder or their sibling) older than 18 years of age will also be excluded. We plan to include grey literature, but will exclude systematic-type reviews, meta-analyses and case studies, conference proceedings/abstracts, books, and book chapters, as well as research in progress. Finally, we will exclude studies published before 2011, given that this evidence has been reviewed previously [6–8] and to provide a more contemporary scope of potential existing resources designed to address the needs of siblings.

We will use a comprehensive search strategy that includes database-specific subject headings and natural language keywords. The databases to be searched include OVID MEDLINE, OVID EMBASE, CINAHL Complete, Proquest Nursing and Allied Health, PsycINFO (via APA platform), Proquest Sociology Collection and Web of Science Core Collection and Proquest Theses and Dissertations. We will modify our search strategy for Google to retrieve grey literature and will also directly search the websites of organizations that produce reports relevant to this topic.

A librarian (E. Yates) developed a comprehensive search strategy to incorporate key concepts, subject headings (MeSH), synonyms and related terms, and search syntax. Text words contained in the titles and abstracts of relevant articles and the index terms used to describe the articles were used to inform a full search strategy for OVID MEDLINE. The search strategy, including all identified keywords and index terms, will be adapted for each included database and/or information source. Since all studies that address research question 2 must also meet criteria for research question 1, no additional search terms specific to research question 2 need to be added, given that these studies will already be captured by the search. The reference lists of key included sources of evidence will be screened for additional studies. The draft search strategy was submitted to the Peer Review of Electronic Search Strategies (PRESS) forum on November 25, 2021. The revised search, incorporating the peer reviewers’ suggestions, for one key database (OVID MEDLINE) is included in S1 File. Our PRISMA-ScR checklist is found in S2 File.

### Stage 3: Selecting studies

Stage three describes the process for identifying studies included in the review. We will use Zotero citation management software to manage and organize citations for studies retrieved by the search. After removing duplicates, all publications identified from the search will be uploaded to Covidence Systematic Review Software. The titles and abstracts of each will be screened by two senior graduate students in Covidence. Based on our eligibility criteria described in stage 2, both screeners will label each article as “yes-include” “no-remove” or “maybe”. In Covidence, reviewers can also add study tags to each article. Studies that are labelled as “yes”, or “maybe” will also be labelled with a tag denoting “research question 1” or “research question 2” thus enabling us to identify and extract articles specific to each question. The screeners will be blinded to each other’s responses. Articles both labelled “yes” will be included in the full-text review. Articles that were labelled “maybe” by one or both screeners, as well as other discrepancies between screeners will be flagged and discussed until a consensus is reached. A third reviewer (JEK) will be consulted if screeners cannot reach a consensus. Reasons for exclusion at full-text screening that do not meet the inclusion criteria will be recorded and reported in the scoping review. Following the screening stage, each article will undergo a full-text review for final inclusion in the scoping review.

### Stage 4: Charting the data

Stage 4 of the Arksey and O’Malley Framework provides guidelines on charting data from studies selected for inclusion in our scoping review. Two team members will categorize 10% of the studies included for full-text review (i.e., to determine if all relevant information can be charted in 10% of all studies). The team will discuss disagreements and subsequent edits to our charting framework will be considered. After completing this pilot test of the review, two team members will categorize the information from all included full texts.

### Stage 5: Collating, summarizing, and reporting the results

**Fig 1:**
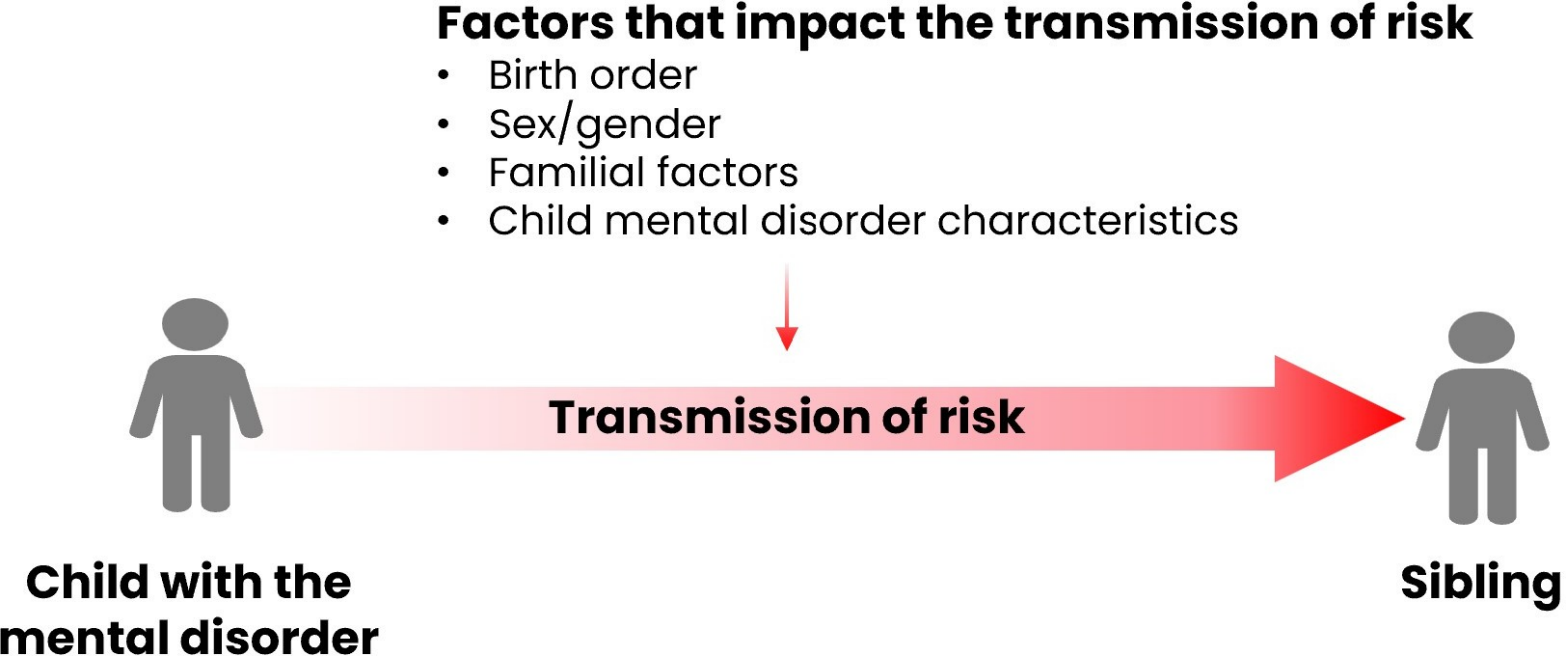
The transmission of risk from the child in the family with a mental disorder to their siblings.

Data from studies that aim to address research question one of this review will be charted in Table 1. Our charting framework in Table 1 is designed to examine how a mental disorder in a child might increase risk in their sibling (Fig 1). Therefore, for each study, we will first chart the child’s mental disorder, then chart other salient factors (i.e., sex, gender and birth order of either child, familial factors, and characteristics of the child’s mental disorder), if examined. Finally, we will chart data on the sibling’s mental health outcome. This framework will enable us to map evidence from studies that accounted for salient factors when examining the impact of child mental illness on sibling mental health. Therefore, we plan to use our charting framework in Table 1 to address each of sub-questions of research question 1. In the results section of our proposed review, we will first comment on evidence examining sibling mental health in the context of age, sex and birth order (sub-question 1). Next, we will discuss evidence on the impact of familial factors on sibling mental health (sub-question 2). Finally, we will summarize evidence on the impact of characteristics of child mental illness (i.e., meltdowns, aggression) on siblings (sub-questions 3). Taken together, this method will provide a comprehensive understanding of sibling mental health in the context of important factors, enable us to identify gaps in the field, and determine whether and how to appropriately target interventions for siblings based on these factors.

**Table 1:** Effects of child mental disorders on their siblings.

Author	Year of publication	Sample size	Child mental disorder	Age/birth order^a^		Sex/gender		Familial factors^b^	Child mental disorder characteristics^c^	Sibling outcome
				Child with mental disorder	Sibling	Child with mental disorder	Sibling			
										

^a^Is the healthy sibling older or younger than the child with the mental disorder.

^b^ Examples include the number of children in the family, two-parent vs. single-parent, parent mental disorders, socioeconomic status.

^c^ Examples include behavioural meltdowns, aggression, depressive episodes.

Data relevant to research question two will be charted in Table 2. The framework in Table 2 will enable us to summarize important evidence from existing interventions. This will provide further important evidence foundation to our larger project, which aims to design an effective resource for siblings of children with mental illness.

**Table 2:** Resources designed to address the needs of siblings of children with a mental disorder.

Author	Year of publication	Sample size	Resource/Intervention type and goals	Study design	Sample characteristics	Healthy sibling outcome	Key findings
							

### Stage 6: Consultation and stakeholder engagement exercise

The proposed scoping review represents a foundational step in a larger project aiming to develop an effective resource for the siblings of children with mental disorders. Therefore, our project will take an integrative knowledge translation approach (iKT) [15]. This approach applies knowledge translation principles to the entire project and requires that knowledge users are involved in each step of the research process [15]. Therefore, the proposed scoping review will involve stakeholders at Children’s Mental Health Ontario (CMHO). Engaging stakeholders throughout the research process is critical to ensure that the project is relevant to knowledge users and to ensure swift uptake. For example, based on these initial stakeholder meetings, parents identified the importance of attempting to extract any data on characteristics of child mental disorders (e.g., behavioural meltdowns, depressive episodes) that may have a particularly salient impact on sibling mental health. Additionally, these stakeholders will provide important insights into efforts made by organizations to address the needs of siblings and provide ideas on how to improve these programs/ expand the scope of these resources. Overall, stakeholders have identified and prioritized the need to identify and develop effective supports/resources for the siblings of children with mental disorders, expressed considerable worry about the health of siblings, and strongly advocated for the development of sibling supports. Therefore, we will consult these stakeholders throughout the scoping review process.

## Conclusion

The siblings of children with mental disorders are at increased risk for psychopathology. Despite evidence suggesting that these siblings experience elevated levels of psychological distress and that parents have expressed concern about siblings, sibling needs are often unmet. This protocol outlines the methodology for a scoping review that aims to provide a comprehensive understanding of the extant literature on the impact that child mental disorders have on their sibling(s), including the role that salient contextual factors (i.e., age, sex, birth order, familial factors, child mental disorder characteristics) might play in these links. We also aim to scope the evidence on potential resources/interventions designed to specifically address the needs of siblings of children with mental disorders. It will examine studies and interventions published after 2011 to ensure that our review is a contemporary assessment of the state of this literature. This method will also provide the foundation needed to develop, enhance, or expand the scope of existing interventions. This scoping review protocol was developed in keeping with gold standard scoping review methodological criteria. Finally, our inclusion of the consultation and stakeholder engagement exercise will ensure that this work is relevant and that results can be rapidly translated to knowledge users. Taken together, this proposed scoping review aims to provide evidence and guidance critical to investing in the health of siblings of children with mental disorders.

## Supporting information

S1 File (DOCX)

S2 File PRISMA-ScR checklist. (DOCX)
