# Supplementary material for: Examining the mental health of siblings of children with a mental disorder: A scoping review protocol
Source: PLoS One. 2022 Sep 15;17(9):e0274135. doi: 10.1371/journal.pone.0274135 (PMC9477329; doi:10.1371/journal.pone.0274135)
Supplement: S1 File — (DOCX) [file pone.0274135.s001.docx]

**Supplemental Material: Examining the mental health of siblings of children with a mental disorder: A scoping review protocol**

John E. Krzeczkowski^1*^ , Terrance J. Wade^2^, Brendan F. Andrade^3,4^, Dillon Browne^5^, Busra Yalcinoz-Ucan^5^, Negin A. Riazi^2^., Elizabeth Yates^6^, Andrea Tagalakis^7^, Karen A. Patte, PhD^2^.

^1^ Department of Psychology, York University, Toronto, Ontario, Canada

^2^ Department of Health Sciences, Brock University, St. Catharines, Ontario, Canada

^3^ Margaret and Wallace McCain Centre for Child, Youth and Family Mental Health, Centre for Addiction and Mental Health, Toronto, Ontario, Canada

^4^Department of Psychiatry, University of Toronto, Toronto, Ontario, Canada

^5^ Department of Psychology, University of Waterloo, Waterloo, Ontario, Canada

^6^ Brock University Library, Brock University, St. Catharines, Ontario, Canada

^7^ Family Partnership Program, Children’s Mental Health Ontario, Toronto, Ontario, Canada

*Corresponding author

Email: [krzeczkj@yorku.ca](mailto:krzeczkj@yorku.ca) (JK)

**OVID MEDLINE search**

Ovid MEDLINE(R) and In-Process, In-Data-Review & Other Non-Indexed Citations <1946 to February 17, 2022>

1 exp Mental Health/ or exp Adaptation, Psychological/ 182352

2 ("mental hygiene" or (mental adj3 (health or hygiene or status)) or (well adj3 being) or (adaptation adj3 psychological)).tw. 275444

3 1 or 2 408180

4 exp Child/ or exp Adolescent/ 3229649

5 (child or Adolescen* or Teen* or Youth* or "Young adult*").tw. 774141

6 4 or 5 3477287

7 3 and 6 113160

8 exp Siblings/ or exp Sibling Relations/ 15032

9 (Sibling* or brother* or sister*).tw. 101155

10 8 or 9 104073

11 Mental Disorders/ 171979

12 ((Mental or behaviour or behavior or psychiatric) adj3 (illness* or disorder* or diagnosis*)).tw. 149700

13 11 or 12 272128

14 Anxiety Disorders/ 37836

15 anxiet*.tw. 219598

16 14 or 15 227721

17 Agoraphobia/ 2645

18 Agoraphobia*.tw. 3156

19 exp Neurotic Disorders/ 17992

20 (neurotic or neuroses or psychoneuroses).tw. 9551

21 17 or 18 4113

22 19 or 20 23085

23 Obsessive-Compulsive Disorder/ 15607

24 ("Obsessive compulsive disorder" or OCD or (Obsessive adj3 compulsive) or anankastic).tw. 20149

25 23 or 24 24322

26 Panic Disorder/ 7150

27 ((Panic adj3 disorder*) or (Panic adj3 attack*)).tw. 11811

28 26 or 27 13074

29 exp Phobic Disorders/ 11927

30 ((Phobic adj3 disorder*) or Phobia*).tw. 9594

31 29 or 30 16734

32 ("social phobia*" or "social anxiety").tw. 9830

33 Bipolar Disorder/ 43187

34 (bipolar or depress*).tw. 547533

35 33 or 34 558574

36 (("impulse control" adj3 disorder*) or (explosive adj3 disorder*)).tw. 1921

37 Firesetting behavior/ 458

38 ((Fire adj3 setting) or Arson or Pyromania*).tw. 560

39 Trichotillomania/ 1047

40 (Trichotillomania* or (Hair adj3 pulling)).tw. 1399

41 Dissociative Identity Disorder/ or Dissociative Disorders/ 4579

42 (Dissociative adj3 (hysteria or reaction or Disorder*)).tw. 1533

43 41 or 42 5179

44 36 or 37 or 38 or 39 or 40 4175

45 ((Eating adj3 disorder*) or Anorexia).tw. 48816

46 Anorexia Nervosa/ 13748

47 45 or 46 50409

48 Avoidant Restrictive Food Intake Disorder/ 117

49 (food adj3 avoidant).tw. 277

50 48 or 49 329

51 Binge-Eating Disorder/ 1911

52 (binge adj3 eating).tw. 6330

53 51 or 52 6529

54 Bulimia Nervosa/ 2601

55 bulimia.tw. 7718

56 54 or 55 8391

57 exp Mood Disorders/ 130413

58 (mood adj3 disorder*).tw. 22587

59 57 or 58 143535

60 exp Depressive Disorder/ 116590

61 ((depressive adj3 disorder*) or depress*).tw. 499509

62 60 or 61 520127

63 (dysthymia or (dysthym* adj3 disorder*)).tw. 2922

64 (PMDD or (premenstrual adj3 dysphor*)).tw. 1101

65 ("Seasonal Affective Disorder" or ((depression or mood) adj3 seasonal)).tw. 1566

66 cyclothym*.tw. 992

67 63 or 64 or 65 or 66 6464

68 exp Neurodevelopmental Disorders/ 197664

69 "Neurodevelopmental Disorder*".tw. 12106

70 ("Attention deficit disorder" or ADHD or ADDH or hyperactivity or (attention adj3 deficit)).tw. 57277

71 "conduct disorder*".tw. 4801

72 (child* and (behavio?rs adj3 disorder*)).tw. 499

73 ("neurotic disorder*" or neuroses).tw. 3559

74 68 or 69 or 70 or 71 or 72 or 73 235959

75 exp Personality Disorders/ 43434

76 ("personality disorder*" or (avoidant adj3 personality) or (narcissistic adj3 disorder)).tw. 21156

77 ((anti?social adj3 (personalit* or behav* or disorder*)) or (dyssocial and behav*) or socipathic or psychopathic).tw. 9329

78 ("Borderline Personality Disorder" or BPD or (borderline adj3 disorder*)).tw. 14622

79 ("Compulsive Personality Disorder" or (compulsive adj3 (personality or disorder*)) or OCD or obsessive compulsive).tw. 20630

80 (dependent adj3 (personality or disorder*)).tw. 1919

81 (histrionic adj3 (personality or disorder*)).tw. 288

82 (paranoid adj3 (personality or disorder*)).tw. 636

83 ((passive-aggressive or negativistic) adj3 (personality or disorder*)).tw. 103

84 (schizoid adj3 (disorder* or personality)).tw. 349

85 (Schizotypal adj3 (Personality or Disorder*)).tw. 1652

86 75 or 76 or 77 or 78 or 79 or 80 or 81 or 82 or 83 or 84 or 85 82243

87 Affective Disorders, Psychotic/ 2303

88 (affective adj3 (disorder* or psychotic or psychos*)).tw. 20564

89 87 or 88 22046

90 Paranoid Disorders/ 4195

91 paranoi*.tw. 8451

92 90 or 91 10638

93 exp Psychotic Disorders/ 55756

94 ((psychos* adj3 disorder) or schizoaffective).tw. 7607

95 93 or 94 59531

96 Psychoses, Substance-Induced/ 5365

97 (psychos* adj3 (drug or substance)).tw. 2120

98 96 or 97 7220

99 Psychoses, Alcoholic/ 2290

100 (psychos* adj3 alcohol).tw. 754

101 99 or 100 3014

102 Schizophrenia/ 106792

103 Schizophren*.tw. 128898

104 102 or 103 149528

105 Somatoform Disorders/ 9515

106 ((Somatoform adj3 Disorder*) or "briquet syndrome").tw. 2306

107 105 or 106 10530

108 Body Dysmorphic Disorders/ 1171

109 (Body adj3 (Dysmorph* or image)).tw. 13285

110 108 or 109 13640

111 substance-related disorders/ 101121

112 ((substance or drug) adj3 (abuse or dependence or addiction)).tw. 57515

113 111 or 112 132393

114 Alcohol-Related Disorders/ 5575

115 (alcohol adj3 (disorder* or abuse or dependence or addiction)).tw. 43756

116 114 or 115 46827

117 Amphetamine-Related Disorders/ 3397

118 (Amphetamine adj3 (disorder* or abuse)).tw. 582

119 117 or 118 3853

120 ((amphetamine or cocaine or inhalant or marijuana or narcotic or Phencyclidine) adj3 (disorder* or abuse)).tw. 5572

121 exp Stress Disorders, Traumatic/ 41769

122 (traumatic adj3 (stress or disorder*)).tw. 19357

123 121 or 122 50330

124 exp Autism Spectrum Disorder/ 36785

125 (autism or autistic or asperger*).tw. 55558

126 124 or 125 58963

127 exp Neoplasms/ 3622793

128 (cancer or neoplasm*).tw. 1998179

129 127 or 128 4135243

130 13 or 16 or 21 or 22 or 25 or 28 or 31 or 35 or 43 or 44 or 47 or 50 or 53 or 56 or 59 or 62 or 67 or 74 or 86 or 89 or 92 or 95 or 98 or 101 or 104 or 107 or 110 or 113 or 116 or 119 or 123 1491305

131 10 and 130 10999

132 126 or 129 4193067

133 7 and 131 819

134 133 not 132 616
